# Association Between Physical Activity Timing and Metabolic Syndrome in Korea: A Functional Principal Component Approach

**DOI:** 10.3390/healthcare13121384

**Published:** 2025-06-10

**Authors:** Suah Park, Hee-Jung Jee

**Affiliations:** Department of Information Statistics, Chungbuk National University, Cheongju 28644, Republic of Korea; 2025221010@chungbuk.ac.kr

**Keywords:** metabolic syndrome, temporal pattern, functional principal component analysis

## Abstract

**Background**: Metabolic syndrome (MetS), characterized by the co-occurrence of obesity, hypertension, hyperglycemia, and dyslipidemia, substantially increases the risk of cardiovascular disease and type 2 diabetes. In South Korea, the prevalence of MetS is steadily increasing. While physical activity is known to mitigate this risk, recent evidence suggests that the timing of activity, not just its volume, may also be important. **Methods**: We analyzed accelerometer data from Korean adults who participated in the 2014–2016 Korea National Health and Nutrition Examination Survey (KNHANES). Functional principal component analysis (FPCA) was applied to minute-level physical activity trajectories to extract key temporal patterns. Logistic regression models assessed associations between the resulting principal component (PC) scores and MetS, adjusting for demographic, behavioral, and occupational factors, as well as total moderate-to-vigorous physical activity (MVPA). **Results**: Among the four extracted components, the third principal component (PC3)—reflecting higher morning and evening activity with reduced afternoon variability—was significantly associated with increased risk of MetS in the fully adjusted model (adjusted OR = 1.117; 95% CI: 1.003–1.244). **Conclusions**: These findings suggest that temporal patterns of physical activity, particularly reduced variability in the afternoon, may be linked to adverse metabolic outcomes. Beyond overall activity volume, the timing and distribution of daily physical activity should be considered in metabolic health research and interventions.

## 1. Introduction

Regular physical activity provides numerous health benefits, including enhanced physical fitness and the prevention or alleviation of various physical and mental disorders [1,2]. According to the Physical Activity Guidelines for Koreans, as issued by the Ministry of Health and Welfare [3], adults aged 19 to 64 are recommended to engage in 150–300 min of moderate-intensity aerobic physical activity per week, or 75–100 min of vigorous-intensity aerobic activity, while minimizing sedentary behavior throughout the day.

However, survey data from the Korea National Health and Nutrition Examination Survey (KNHANES) indicate that compliance with physical activity guidelines has been steadily declining. According to one analysis, as of 2017, approximately 66% of Korean adults did not meet the recommendations for either aerobic or muscle-strengthening exercises, a significant increase from previous years [4]. In particular, the proportion of physically inactive adults increased from 24.6% in 2008 to 42.9% in 2014, highlighting a concerning trend of insufficient physical activity across diverse demographic groups and contributing to widening health disparities.

Physical activity is known to reduce the risk of cardiovascular disease and type 2 diabetes, and it plays a key role in the prevention of obesity and metabolic syndrome (MetS) [5,6,7,8]. MetS is characterized by the coexistence of risk factors, such as abdominal obesity, hypertension, hypertriglyceridemia, low high-density lipoprotein (HDL) cholesterol, and impaired fasting glucose. This condition significantly increases the risk of cardiovascular disease and type 2 diabetes. In South Korea, the prevalence of MetS has been rising steadily, with over 30% of adults diagnosed in 2023 [1]. Although MetS is influenced by a combination of genetic, metabolic, and behavioral factors, physical inactivity is widely recognized as a major contributor.

Regular exercise is considered essential for both the prevention and management of MetS. Research shows that people who exercise regularly are at a lower risk of developing MetS compared to those who are not physically active [8,9]. A meta-analysis of studies including a total of 39 randomized controlled trials (RCTs) and 2132 participants demonstrated that exercise training significantly improves all MetS risk factors [10].

Although numerous studies [5,11,12,13,14,15,16] have examined the association between physical activity and metabolic syndrome (MetS), most have focused primarily on the total volume and intensity of activity. However, recent findings [17,18,19] suggest that the timing of physical activity throughout the day may also play a significant role in shaping health outcomes. In particular, physical activity performed at different times of day may induce distinct physiological responses, potentially influencing the risk of developing MetS.

The aim of this study was to investigate whether the timing of physical activity is associated with metabolic syndrome in Korean adults, controlling for moderate-to-vigorous physical activity levels and other potential risk factors. Using data from the 2014–2016 KNHANES accelerometer dataset, we applied functional principal component analysis (FPCA) to identify patterns in daily physical activity over time. By analyzing both when and how intensely people were physically active, this study takes a multidimensional approach to better understand how physical activity patterns affect metabolic health.

## 2. Materials and Methods

### 2.1. Data Source and Study Design

The Korea National Health and Nutrition Examination Survey (KNHANES) is a nationally representative survey conducted annually under the National Health Promotion Act to assess the health and nutritional status of the Korean population. Approximately 10,000 individuals are sampled each year. The survey was first introduced in 1998 and conducted every three years until 2007, after which it has been implemented annually.

In the earlier phases of the survey, physical activity data were collected exclusively through self-reported questionnaires. Starting in 2014, objective measurements using accelerometers were added to complement the self-reported data. This approach was intended to address the limitations of self-reported methods, including recall bias and limited accuracy, and to enable more precise quantification of physical activity intensity and temporal patterns.

Specifically, the ActiGraph GT3X+ accelerometer was used to collect triaxial acceleration data [20]. This device measures acceleration across three axes: vertical (axis 1), anteroposterior (axis 2), and mediolateral (axis 3). For the purpose of physical activity intensity analysis in KNHANES, the vertical axis (axis 1) was primarily used [21].

Participants were instructed to wear the accelerometer on their waist for seven consecutive days, except during activities such as swimming, showering, and sleeping. The device was configured to begin recording data at midnight after informed consent was obtained.

### 2.2. Study Participants

This study utilized data from the 2014–2016 Korea National Health and Nutrition Examination Survey (KNHANES), including both accelerometer-based physical activity data and health examination data. Although the accelerometer survey in KNHANES was conducted from 2014 to 2017, the 2017 data were excluded because that cycle included only older adults, aged 65 and above. This exclusion was made to ensure consistency in the age range of the study population.

Participants were eligible for inclusion in the study if they (1) were part of the 2014–2016 KNHANES accelerometer subsample; (2) had at least three valid days of accelerometer wear-time with a minimum of 10 h per day, as defined by the criteria proposed by Troiano [22]; and (3) had complete data on relevant health examination and demographic variables.

**Figure 1:**
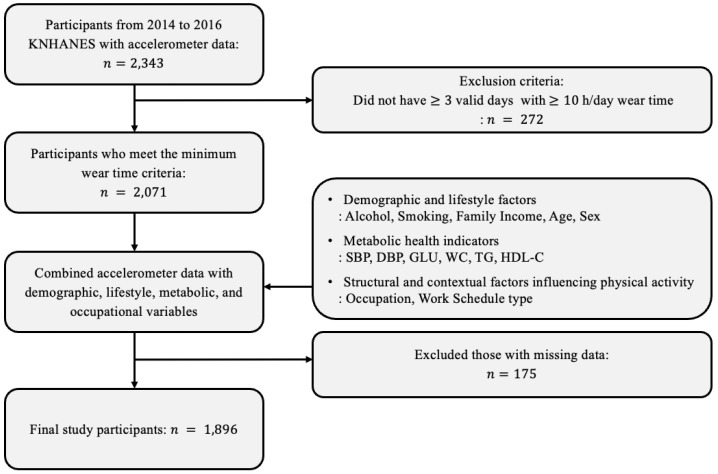
Flowchart of study participant selection from the 2014–2016 Korea National Health and Nutrition Examination Survey (KNHANES) accelerometer dataset.

A total of 2343 individuals from the 2014–2016 accelerometer dataset were initially considered. Among them, 272 participants were excluded for not meeting the minimum wear-time requirement. Of the remaining 2071, an additional 175 individuals were excluded due to missing covariate information. As a result, the final analytic sample comprised 1896 participants. A detailed flowchart of the participant selection process is provided in Figure 1.

### 2.3. Physical Activity

Physical activity intensity was measured using the variable PAXINTEN, which represents the minute-by-minute recorded activity intensity. Each participant provided seven days of data recorded at one-minute intervals, resulting in a total of 10,080 rows per individual.

For the accelerometer data, the PAXDAY variable (day of the week) varied depending on the start date of data collection. However, under the assumption that individuals maintain consistent daily activity patterns, PAXDAY values were adjusted to ensure that all participants’ data began on Sunday. This adjustment was made to systematically analyze temporal variations in physical activity patterns and ensure consistency across the participants’ data.

In addition to temporal pattern analysis, we also calculated total moderate-to-vigorous physical activity (MVPA) time to classify participants by overall activity levels. MVPA was defined based on PAXINTEN thresholds, where moderate-intensity activity corresponded to values between 2020 and 5998, and vigorous-intensity activity to values of 5999 or higher. Only bouts sustained for at least 10 consecutive minutes were considered, allowing for a 2 min drop below the threshold within the window. Weekly MVPA duration was then used to classify participants into four groups in accordance with WHO guidelines [23]:

(1) Inactive: 0 min/week;
(2) Insufficiently active: 1–149 min/week;
(3) Active: 150–299 min/week;
(4) Highly active: ≥300 min/week.

These categories are hereafter referred to as the activity group, and are included as a covariate in the regression models to account for overall physical activity volume.

### 2.4. Diagnostic Criteria for Metabolic Syndrome

The MetS status was generated as the outcome variable using key health indicators from the health examination data to determine the presence of MetS. A MetS diagnosis was considered in patients meeting at least three of the following criteria: high waist circumference, high triglycerides, low HDL cholesterol, high blood pressure, and high fasting glucose.

These criteria are based on the NCEP-ATP III (National Cholesterol Education Program Adult Treatment Panel III) [24], with the waist circumference thresholds modified for Asian populations according to guidelines from the Korean Society for the Study of Obesity [25]. For example, the waist circumference threshold was set at ≥90 cm for men and ≥85 cm for women. Triglycerides were considered elevated if ≥150 mg/dL, and HDL cholesterol was classified as low if <40 mg/dL for men and <50 mg/dL for women. Blood pressure was defined as elevated if ≥130/85 mmHg or if the individual was undergoing treatment. Fasting glucose was defined as elevated if ≥100 mg/dL or if the individual was receiving treatment. The resulting MetS variable was recorded as a binary indicator: 0 (absence of MetS) or 1 (presence of MetS).

### 2.5. Confounding Factors

To analyze the association between metabolic syndrome and physical activity, potential confounding factors, including age, sex, family income, occupation type, work schedule type, smoking status, and alcohol consumption, were incorporated into the analysis. These variables were selected based on the prior literature to ensure that the relationship between metabolic syndrome and physical activity was not confounded by external factors [26]. In particular, occupation type and work schedule type were included as confounders, as prolonged occupational sedentary behavior and irregular work schedules have been reported to affect cardiometabolic health outcomes in recent studies [27].

Age was treated as a continuous variable, and sex was coded as a binary variable (male or female). Family income was categorized into quartiles based on average monthly income. Occupation type was classified into three categories according to previous research: non-manual worker (e.g., managers, professionals, office workers), manual worker (e.g., service/sales workers, skilled agricultural and fishery workers, machine operators, laborers), and economically inactive individuals (e.g., military personnel, students, homemakers) [28]. Work schedule type was categorized into day shift, evening/night shift (evening or night shifts), rotating shift (regular day and night shifts, 24 h shifts, irregular shifts), other (e.g., split shifts), and not employed.

Smoking status was categorized into three groups: non-smoker, former smoker, and current smoker. Alcohol consumption was classified based on the frequency of drinking during the past year as follows: not applicable, non-drinker, 1 or less times/week, 2–4 times/month, 2–3 times/week, and 4 or more times/week.

All these variables were adjusted for in the multivariable models to control for their potential confounding effects.

## 3. Statistical Analysis

### 3.1. Participant Characteristics by Metabolic Syndrome Status

Statistical analyses were performed using R version 4.4.2. The distribution of continuous data was analyzed using histograms and the Shapiro–Wilk test. Continuous data with a normal distribution were expressed as the mean and standard deviation, and continuous data for which a normal distribution could not be assumed were expressed as the median (IQR). Categorical data were expressed as frequency (percent). To compare characteristics between participants with and without metabolic syndrome, appropriate statistical tests were applied depending on variable type and distribution. We performed independent-variable *t*-tests for continuous variables that met normality, Wilcoxon rank-sum tests for continuous variables that did not meet normality, and chi-square tests for categorical variables. If the expected frequency in any cell was less than 5, Fisher’s exact test was used instead of the chi-square test.

### 3.2. Functional Principal Component Analysis of Physical Activity

To capture individual-level temporal patterns of physical activity, we applied functional principal component analysis (FPCA) to 7-day minute-level accelerometer data (10,080 time points per participant). Prior to FPCA, each participant’s activity trajectory was smoothed using a B-spline basis with 65 basis functions. The smoothing parameter was set to *λ* = 10^−6^ to retain short-term variation while minimizing overfitting. This approach allowed us to represent each person’s daily activity as a continuous function of time.

FPCA was then conducted on the smoothed curves to extract orthogonal eigenfunctions, representing dominant temporal patterns in physical activity. Because the eigenfunctions are orthogonal by construction, the resulting principal component scores are uncorrelated and can be interpreted as independent dimensions of activity timing. We retained the first four principal components, which collectively explained the majority of variability in the daily activity profiles.

These FPCA scores were used as independent variables in multivariable logistic regression models to assess their associations with the presence of MetS. All models were adjusted for age, sex, family income, smoking status, alcohol consumption, occupation type, work schedule type, and average daily moderate-to-vigorous physical activity (MVPA).

All statistical analyses were conducted using R version 4.4.2 [29]. Functional data analysis was performed using the fda package [30].

### 3.3. Association Between Functional Principal Component Scores and Metabolic Syndrome

Further analyses were conducted in order to identify the association between various patterns of physical activity and metabolic syndrome. Logistic regression analyses were performed, with principal component scores obtained through functional principal component analysis and potential confounding factors considered as independent variables, with MetS as the dependent variable. The absence of multicollinearity among the independent variables was confirmed by evaluating their variance inflation factors (VIFs).

## 4. Results

### 4.1. Participant Characteristics

Table 1 presents the comparison of participant characteristics and metabolic syndrome-related factors according to the presence or absence of MetS. All the continuous variables (SBP, DBP, GLU, WC, TG, HDL-C) showed statistically significant differences between groups (all p< 0.001). Significant group differences were also observed in categorical variables such as sex, smoking status, family income, alcohol consumption, occupation type, and work schedule type (all *p* < 0.001), supporting their role as potential confounders. By contrast, no significant difference was found in the distribution of activity groups between MetS and without-MetS individuals (*p* = 0.994). This result may be attributed to the influence of multiple confounding factors, which were not accounted for in the univariable analysis.

### 4.2. Temporal Variation in Physical Activity

**Figure 2:**
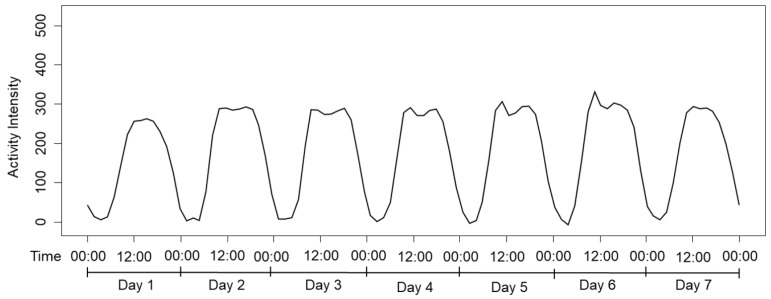
Mean function of minute-level physical activity intensity over seven consecutive days.

FPCA is an analytical method that captures temporal variations and changes in activity patterns, allowing for a more comprehensive summary of the overall variability in the data, while identifying key principal components. Figure 2 shows the mean function of the entire dataset, illustrating average physical activity levels over time. The x-axis represents clock time over seven consecutive days, with the underlying data collected at 1 min intervals. For visual clarity, the axis is labeled at 12 h intervals (e.g., 0:00 and 12:00), while the y-axis reflects the mean minute-level activity intensity averaged across all participants and valid days. This plot reveals periodic activity patterns, providing fundamental insights into the structure of the data and serving as a crucial foundation for the FPCA analysis.

The FPCA results indicate that the overall variability in the data can be divided into four major principal components. The scree plot illustrates that the first principal component (PC1) accounts for approximately 16.48% of the total variance, with the contributions of subsequent components gradually decreasing. This indicates that FPCA effectively summarizes the variability structure of the data and captures the key patterns in physical activity fluctuations.

**Figure 3:**
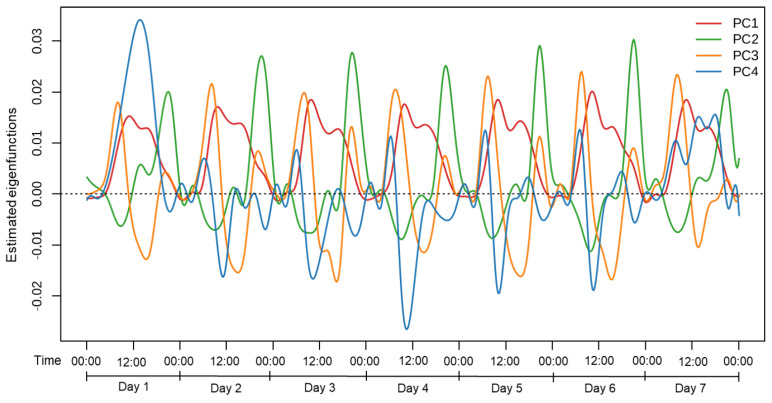
Estimated eigenfunctions (PC1–PC4) from functional principal component analysis of daily physical activity. PC1 reflects overall movement intensity across the day; PC2 captures evening activity variation; PC3 contrasts afternoon activity with morning and evening peaks; and PC4 represents irregular weekend-related fluctuations.

Figure 3 shows the eigenfunctions derived from the FPCA. These eigenfunctions correspond to the principal components and describe the main patterns of variation in daily physical activity. The x-axis represents time in 24 h format, repeated across seven consecutive days, and the y-axis shows the direction and magnitude of deviation from the mean function. The data were collected at 1 min intervals using accelerometers. Positive values indicate increased activity relative to the mean at a given time point, while negative values indicate decreased activity. The main characteristics of each principal component are as follows.

**Figure 4:**
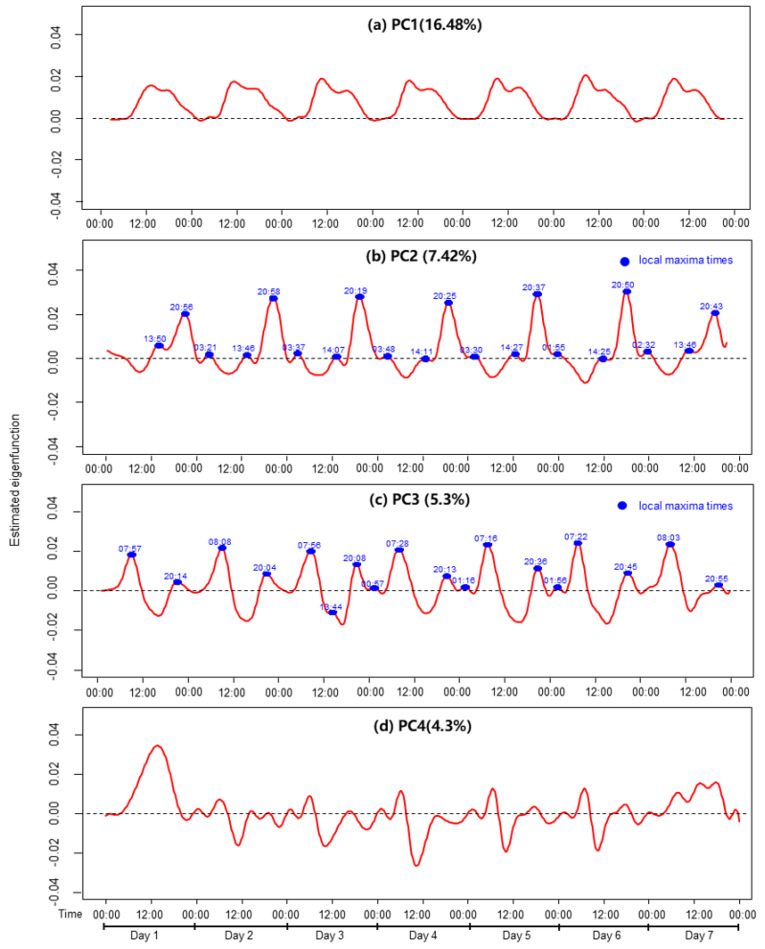
Estimated eigenfunctions of the four principal components (PC1–PC4) from FPCA. Red lines show the estimated eigenfunctions, and blue dots in panels (**b**,**c**) highlight the local maxima, indicating time periods with the greatest variation.

The first principal component (PC1) explains 16.48% of the total variance and primarily reflects the overall level of physical activity across the day. It captures a broad, periodic pattern aligned with the diurnal cycle, with relatively uniform fluctuations in activity intensity over the 24 h period. Individuals with higher PC1 scores tended to engage in more frequent general movement throughout the day. PC1 can encompass a wide range of movements, including rather low-intensity and casual physical activity that is not specifically structured. Thus, PC1 can be interpreted as reflecting overall movement intensity across time. The second principal component (PC2) accounts for an additional 7.42% of the variance and primarily reflects variations in activity levels during the evening. The local maxima, indicated by blue dots, represent a sharp increase in activity levels during this period, with the peak values concentrated between 20:00 and 21:00. This suggests that evening activity levels are the key factors influencing PC2. The third principal component (PC3) explains an additional 5.3% of the variance and is characterized by a clear bimodal pattern, with local maxima occurring consistently around 07:00–08:00 and 20:00–21:00, as shown in Figure 4c. This suggests that individuals with higher PC3 scores tended to engage in physical activity primarily during these two time windows, while exhibiting relatively less variation in activity during the afternoon hours (12:00–18:00). Conversely, lower PC3 scores may indicate more evenly distributed or afternoon-concentrated activity. Therefore, PC3 likely captures the extent to which an individual’s daily activity pattern avoids or emphasizes afternoon activity. Finally, the fourth principal component (PC4) explains 4.3% of the additional variance and appears to be associated with changes in activity levels on weekends (Figure 4).

### 4.3. Association Between Principal Component Scores and Metabolic Syndrome

The principal component scores extracted for each individual were standardized and used as independent variables to evaluate their association with metabolic syndrome (MetS) using logistic regression analysis.

Four logistic regression models were sequentially constructed to examine the robustness of the associations.

(1) Model 1: A simple logistic regression model including only the FPCA scores (PC1–PC4) as independent variables to estimate unadjusted odds ratios.
(2) Model 2: Adjusted for key demographic confounding variables—age, sex, family income, alcohol consumption, and smoking status.
(3) Model 3: Further included occupation type and work schedule type—in addition to model 2.
(4) Model 4: Additionally included MVPA-based activity groups (i.e., inactive, insufficiently active, active, and highly active) to account for physical activity volume.

The associations between PC1–PC4 and MetS across all models are summarized in Table 2. Estimates for the confounding factors included in models 2–4 are presented separately in Supplementary Table S2.

In model 1, none of the FPCA components were significantly associated with MetS. PC3 showed a marginal effect (unadjusted OR = 1.061, 95% CI: 0.963–1.169, *p* = 0.232), but this did not reach statistical significance. However, after adjusting for demographic factors in model 2, PC3 was significantly associated with MetS (adjusted OR = 1.105, 95% CI: 0.993–1.230, *p* = 0.067). This association persisted in model 3 after the inclusion of occupation type and work schedule type (adjusted OR = 1.118, 95% CI: 1.004–1.246, *p* = 0.042), and remained significant in model 4 with additional adjustment for activity group based on MVPA (adjusted OR = 1.117, 95% CI: 1.003–1.244, *p* = 0.044). These findings suggest that low variation in afternoon activity (captured by PC3) is independently associated with higher odds of MetS. By contrast, PC1, PC2, and PC4 remained non-significant across all models (*p* > 0.200), indicating a limited relationship with MetS.

Among the confounding variables, age consistently showed a strong and significant association with metabolic syndrome in all models. For example, in model 4, for every 1 year increase in age, the odds of metabolic syndrome increased by approximately 6% (adjusted OR = 1.061, 95% CI: 1.050–1.073, p< 0.001). Gender was also significant in all models, with female participants having lower odds of metabolic syndrome than male participants (model 4: adjusted OR = 0.403, 95% CI: 0.300–0.543, *p* < 0.001). Alcohol consumption was not significantly associated with metabolic syndrome in most frequency categories, but those who drank four or more times a week had significantly higher odds of metabolic syndrome compared to non-drinkers (model 4: adjusted OR = 1.713, 95% CI: 1.017–2.885, *p* = 0.043). This suggests that frequent drinking may potentially increase the risk of metabolic syndrome. Family income in the third quartile was significantly associated with a lower probability of MetS, especially from model 3 onwards (model 3: adjusted OR = 0.654, 95% CI: 0.462–0.928, *p* = 0.017; model 4: adjusted OR = 0.660, 95% CI: 0.464–0.938, *p* = 0.026).

In terms of occupation type, the economically inactive population had a significantly higher probability of MetS than non-manual workers in both models 3 and 4 (model 3: adjusted OR = 1.911, 95% CI: 1.283–2.847, model 4: adjusted OR = 1.958, 95% CI: 1.312–2.922, *p* < 0.001). In contrast, there was no significant difference with manual workers.

Interestingly, evening/night-shift workers had a lower probability of MetS than day-shift workers (model 4: adjusted OR = 0.617, 95% CI: 0.404–0.943, *p* = 0.026). This result should be interpreted cautiously. One potential explanation is that people with irregular working hours may be more likely to engage in physical activity in the afternoon or early evening. This is the time period identified as potentially protective in FPCA (see PC3). However, unmeasured confounders such as sleep patterns or circadian rhythm disturbances cannot be ruled out.

Regarding physical activity, model 4 included a categorical group based on MVPA. Only the highly active group had a significantly lower probability of developing the metabolic syndrome (MetS) (adjusted OR = 0.453, 95% CI: 0.240–0.853, *p* = 0.014). In contrast, no significant difference was observed in the insufficiently active or active groups. To complement this analysis, MVPA was additionally assessed as a continuous variable (see Supplementary Table S3). This result supported the robustness of the main study results, showing a significant negative association between total MVPA and the risk of MetS.

Other variables such as smoking status were not statistically significant in the fully adjusted model. The full regression results for all models and covariates are presented in Supplementary Table S2.

## 5. Discussion

The current findings contribute to a growing body of evidence suggesting that not only the quantity but also the timing of physical activity may influence metabolic health. Our observation of an association between low afternoon activity variability and increased risk of metabolic syndrome (MetS) is consistent with prior studies highlighting the role of circadian timing in metabolic regulation [31]. Recent research further supports this interpretation. For example, Moholdt et al. (2021) reported that evening exercise improved glycemic control and metabolic biomarkers in overweight men [32], and Clavero-Jimeno et al. (2024) found that performing more than half of daily MVPA in the evening was associated with lower 24 h glucose levels [33]. These results suggest that circadian rhythms and hormonal fluctuations may modulate metabolic responses to physical activity depending on the time of day.

Interestingly, participants working evening or night shifts exhibited lower odds of MetS compared to day-shift workers. One possible explanation is that individuals with non-standard work schedules may engage in more physical activity during the afternoon or early evening hours, aligning with the beneficial time window (PC3) identified in our analysis. However, this interpretation warrants caution, as unmeasured confounders—such as sleep timing, circadian disruption, or dietary patterns—could also contribute to this association.

These findings reinforce the central hypothesis of this study—that the timing of physical activity plays a critical role in metabolic health. In particular, individuals with higher variability in afternoon activity showed a reduced risk of MetS, highlighting the importance of not just being active, but being active at specific times of the day. Although the total volume of physical activity is undoubtedly important—only those in the highly active group showed significantly lower odds of MetS (adjusted OR = 0.453, 95% CI: 0.240–0.853 *p* = 0.014)—our results suggest that temporal distribution of activity may further differentiate metabolic outcomes among individuals with similar total activity levels. These findings underscore the need to consider not only how much physical activity is accumulated, but also when it occurs, as part of targeted prevention strategies for MetS.

Future research should investigate whether modifying the timing of physical activity—particularly by increasing afternoon activity variability—can effectively reduce the risk of MetS. Experimental studies incorporating time-targeted physical activity interventions would help determine the causal role of circadian timing. Populations with irregular work schedules may be particularly suitable for such interventions, given their greater exposure to circadian disruption. These efforts could inform the development of more personalized and chronobiologically informed physical activity guidelines for metabolic health.

However, several limitations should be noted. First, given the cross-sectional design of this study, it is difficult to infer causality between temporal physical activity patterns and MetS. It remains unclear whether low afternoon variability increases the risk of MetS, or whether individuals with MetS exhibit different activity rhythms. Second, although the FPCA-based method effectively captured temporal variation in activity, the variance explained was relatively low, suggesting that other behavioral or physiological factors may also play a role. Third, the time interval between the last exercise session and blood sampling was not recorded or controlled, which may have influenced the measurement of serum lipoproteins such as triglycerides and HDL-C. Acute effects of recent physical activity on lipid profiles have been reported in prior studies, and could potentially confound the association between physical activity patterns and metabolic biomarkers [34].

## 6. Conclusions

This study examined the association between temporal patterns of physical activity and metabolic syndrome (MetS) using accelerometer data from a nationally representative sample of Korean adults. Functional principal component analysis was applied to extract key time-specific activity patterns. Notably, lower variability in afternoon physical activity—captured by the third principal component (PC3)—was significantly associated with an increased risk of MetS, independent of total MVPA and other confounders. These findings suggest that not only the volume but also the timing of physical activity may play a critical role in metabolic health. While individuals in the highly active group exhibited a lower risk of MetS, the timing of activity provided additional explanatory power beyond total MVPA. In particular, afternoon activity variability emerged as a relevant marker of metabolic risk. Current physical activity guidelines emphasize duration and intensity, but these results highlight the potential value of incorporating time-specific considerations. Promoting more variable or sustained physical activity in the afternoon may enhance preventive strategies for MetS, especially in populations with irregular activity schedules or disrupted circadian rhythms. Given the cross-sectional nature of this study, longitudinal and experimental research is warranted to clarify causality and explore the physiological mechanisms underlying time-specific activity effects. Such efforts could support the development of personalized, chronobiologically informed physical activity guidelines aimed at improving metabolic health. 

## Figures and Tables

**Table 1:** Demographic and metabolic syndrome-related characteristics of the study participants.

Characteristic	Without MetS (n = 1347)	MetS (n = 549)	Total (n = 1896)	*p*-Value
Age, mean (SD)	41.9 (12.5)	49.4 (9.4)	43.5 (12.3)	<0.001
Sex, no. (%)				
Male	406 (30.1)	306 (55.7)	712 (37.6)	<0.001
Female	941 (69.9)	243 (44.3)	1184 (62.4)	
Family income, no. (%)				
1st quartile	186 (13.8)	109 (19.9)	295 (15.6)	<0.001
2nd quartile	365 (27.1)	176 (32.1)	541 (28.5)	
3rd quartile	420 (31.2)	129 (23.5)	549 (29.0)	
4th quartile	376 (27.9)	135 (24.6)	511 (27.0)	
Smoking status, no. (%)				
Non-smoker	984 (73.1)	281 (51.2)	1265 (66.7)	<0.001
Former smoker	201 (14.9)	163 (29.7)	364 (19.2)	
Current smoker	162 (12.0)	105 (19.1)	267 (14.1)	
Alcohol consumption, no. (%)				
Non-drinker	289 (21.5)	115 (20.9)	404 (21.3)	<0.001
1 or less times/week	492 (36.5)	149 (27.1)	641 (33.8)	
2–4 times/month	342 (25.4)	133 (24.2)	475 (25.1)	
2–3 times/week	176 (13.1)	100 (18.2)	276 (14.6)	
4 or more times/week	48 (3.6)	52 (9.5)	100 (5.3)	
Occupation type, no. (%)				
Non-manual worker	703 (52.2)	204 (37.2)	907 (47.8)	<0.001
Manual worker	554 (41.1)	284 (51.7)	838 (44.2)	
Economically inactive	90 (6.7)	61 (11.1)	151 (8.0)	
Work schedule type, no. (%)				
Day shift	822 (61.0)	402 (73.2)	1224 (64.6)	<0.001
Evening/night shift	150 (11.1)	34 (6.2)	184 (9.7)	
Rotating shift	42 (3.1)	19 (3.5)	61 (3.2)	
Other	5 (0.4)	4 (0.7)	9 (0.5)	
Not employed	328 (24.4)	90 (16.4)	418 (22.0)	
Activity group, no. (%)				
Inactive (0 min/week)	318 (23.6)	129 (23.4)	447 (23.6)	0.994
Insufficiently active (1–149 min/week)	829 (61.5)	340 (61.9)	1169 (61.6)	
Active (150–299 min/week)	150 (11.1)	61 (11.1)	211 (11.1)	
Highly active (≥300 min/week)	50 (3.7)	19 (3.4)	69 (3.6)	
Metabolic syndrome factors				
SBP, mean (SD)	109.3 (11.5)	125.9 (14.5)	114.1 (14.5)	<0.001
DBP, mean (SD)	72.0 (8.0)	82.6 (9.9)	75.1(9.8)	<0.001
GLU, median (IQR)	91.0 (9.0)	104.0 (17.0)	93.0 (13.0)	<0.001
WC, mean (SD)	76.6 (7.9)	88.7 (8.6)	80.1 (9.8)	<0.001
TG, median (IQR)	81.0 (59.0)	171.0 (109.0)	99.0 (89.0)	<0.001
HDL-C, mean (SD)	55.2 (12.6)	46.0 (10.4)	52.5 (12.7)	<0.001

**Note:** SBP: systolic blood pressure; DBP: diastolic blood pressure; GLU: fasting glucose; WC: waist circumference; TG: triglycerides; HDL-C: high-density lipoprotein cholesterol. Definitions of categorical variables (e.g., sex, family income, smoking status, alcohol consumption, occupation type, work schedule type, and activity group) are provided in Section 2.3 and Section 2.5.

**Table 2:** Logistic regression results for the association between FPCA scores and metabolic syndrome (n=1896).

Variable	Model 1		Model 2		Model 3		Model 4	
	Unadjusted OR (95% CI)	*p*-Value	Adjusted OR (95% CI)	*p*-Value	Adjusted OR (95% CI)	*p*-Value	Adjusted OR (95% CI)	*p*-Value
PC1	1.016 (0.921, 1.121)	0.750	1.061 (0.952, 1.182)	0.285	1.048 (0.939, 1.167)	0.406	1.050 (0.941, 1.171)	0.381
PC2	1.004 (0.911, 1.107)	0.930	0.979 (0.881, 1.089)	0.705	0.974 (0.875, 1.084)	0.629	0.976 (0.877, 1.086)	0.650
PC3	1.061 (0.963, 1.169)	0.232	1.105 (0.993, 1.230)	0.067	1.118 (1.004, 1.246)	0.042	1.117 (1.003, 1.244)	0.044
PC4	1.036 (0.939, 1.142)	0.481	1.010 (0.909, 1.123)	0.848	1.009 (0.908, 1.123)	0.856	1.008 (0.906, 1.121)	0.884

Logistic regression analysis results for the association between FPCA scores (PC1–PC4) and metabolic syndrome (n=1896). Four sequential models were fitted. Model 1 included only FPCA scores. Model 2 additionally adjusted for demographic confounders (age, sex, family income, alcohol consumption, and smoking status). Model 3 additionally included occupation type and work schedule type. Model 4 additionally included an MVPA-based physical activity group (activity group) to explain overall activity level. Complete estimates for all confounding factors are presented in Supplementary Table S2.

## Data Availability

The datasets analyzed during the current study are publicly available from the Korea National Health and Nutrition Examination Survey (KNHANES) website: https://knhanes.kdca.go.kr/knhanes/surveyIntr/grtg.do, accessed on 23 August 2024.

## Supplementary Materials

The following supporting information can be downloaded at: https://www.mdpi.com/article/10.3390/healthcare13121384/s1, Table S1: Variance inflation factors (VIFs) for all variables in the logistic regression model; Table S2: Logistic regression results for the association between FPCA scores and metabolic syndrome (n = 1896). Table S3: Logistic regression results for model 4 with MVPA as a continuous variable (n = 1896).
