# Supplementary material for: Association Between Physical Activity Timing and Metabolic Syndrome in Korea: A Functional Principal Component Approach
Source: Healthcare (Basel). 2025 Jun 10;13(12):1384. doi: 10.3390/healthcare13121384 (PMC12192572; doi:10.3390/healthcare13121384)
Supplement: Supplementary file 1 [file healthcare-13-01384-s001.zip › healthcare-3587030-supplementary.pdf]

Supplementary Material for:

Association between Physical Activity Timing and Metabolic Syndrome in Korea: A Functional Principal Component Approach

Table S1. Variance Inflation Factors (VIFs) for all variables in the logistic regression model.

**Variable**	**VIF**
PC1	1.018
PC2	1.015
PC3	1.018
PC4	1.017
Sex	1.824
Age	1.181
Alcohol Consumption	1.343
Smoking Status	1.843
Family Income	1.146
Occupation Type	1.171
Work Schedule Type	1.212
Activity Group	1.097

Note: All VIF values were below 2, indicating no significant multicollinearity among the covariates.

Multicollinearity was assessed by calculating the Variance Inflation Factor (VIF) for all variables included in the logistic regression model 4. The VIF values for FPCA scores (PC1–PC4) and covariates were all below 2.0, suggesting that multicollinearity was not a concern in this model.

Table S2. Logistic regression results for the association between FPCA scores and metabolic syndrome ($n = 1{,}896$).

Variable / Model	Model 1 Unadjusted OR (95% CI)	p-value	Model 2 Adjusted OR (95% CI)	p-value	Model 3 Adjusted OR (95% CI)	p-value	Model 4 Adjusted OR (95% CI)	p-value
PC1	1.016 (0.921, 1.121)	.750	1.061 (0.952, 1.182)	.285	1.048 (0.939, 1.167)	.406	1.050 (0.941, 1.171)	.381
PC2	1.004 (0.911, 1.107)	.930	0.979 (0.881, 1.089)	.705	0.974 (0.875, 1.084)	.629	0.976 (0.877, 1.086)	.650
PC3	1.061 (0.963, 1.169)	.232	1.105 (0.993, 1.230)	.067	1.118 (1.004, 1.246)	.043	1.117 (1.003, 1.244)	.044
PC4	1.036 (0.939, 1.142)	.481	1.010 (0.909, 1.123)	.848	1.009 (0.908, 1.123)	.856	1.008 (0.906, 1.121)	.884
Age	–	–	1.061 (1.050, 1.071)	<.001	1.056 (1.048, 1.065)	<.001	1.061 (1.050, 1.073)	<.001
**Sex**				<.001		<.001		<.001
Male (Ref)	–	–	–	–	–	–	–	–
Female	–	–	0.413 (0.311, 0.549)	<.001	0.760 (0.484, 1.193)	<.001	0.403 (0.300, 0.543)	<.001
**Alcohol Consumption**				.029		.058		.071
Non-drinker (Ref)	–	–	–	–	–	–	–	–
≤ 1 times/week	–	–	0.902 (0.663, 1.228)	.512	0.867 (0.636, 1.185)	.374	0.879 (0.643, 1.201)	.418
2–4 times/month	–	–	1.131 (0.808, 1.583)	.472	1.088 (0.774, 1.246)	.628	1.098 (0.781, 1.544)	.591
2–3 times/week	–	–	1.317 (0.897, 1.934)	.160	1.244 (0.844, 1.834)	.269	1.258 (0.852, 1.857)	.247
≥ 4 times/week	–	–	1.909 (1.141, 3.194)	.014	1.737 (1.033, 2.922)	.037	1.713 (1.017, 2.885)	.043
**Family Income**				.007		.031		.037
1st Quartile (Ref)	–	–	–	–	–	–	–	–
2nd Quartile	–	–	0.904 (0.651, 1.255)	.547	0.941 (0.675, 1.309)	.714	0.942 (0.675, 1.316)	.729
3rd Quartile	–	–	0.609 (0.433, 0.859)	.004	0.654 (0.462, 0.928)	.017	0.660 (0.464, 0.938)	.026
4th Quartile	–	–	0.680 (0.483, 0.957)	.027	0.721 (0.508, 1.025)	.068	0.725 (0.509, 1.032)	.075
**Smoking Status**				.165		.172		.195
Non-Smoker (Ref)	–	–	–	–	–	–	–	–
Former Smoker	–	–	1.342 (0.969, 1.858)	.077	1.312 (0.912, 1.886)	.082	1.345 (0.967, 1.870)	.078
Current Smoker	–	–	1.307 (0.912, 1.874)	.145	1.938 (1.159, 3.239)	.142	1.256 (0.872, 1.809)	.222
**Occupation Type**						.007		.005
Non-manual worker (Ref)	–	–	–	–	–	–	–	–
Manual worker	–	–	–	–	1.128 (0.884, 1.438)	.332	1.118 (0.875, 1.428)	.371
Economically inactive	–	–	–	–	1.911 (1.283, 2.847)	<.001	1.958 (1.312, 2.922)	<.001
**Work Schedule Type**						.088		.106
Day Shift (Ref)	–	–	–	–	–	–	–	–
Evening/Night Shift	–	–	–	–	0.616 (0.404, 0.940)	.025	0.617 (0.404, 0.943)	.026
Rotating Shift	–	–	–	–	0.734 (0.397, 1.361)	.327	0.718 (0.386, 1.336)	.296
Other	–	–	–	–	1.282 (0.300, 5.473)	.737	1.201 (0.280, 5.160)	.805
Not employed	–	–	–	–	0.767 (0.301, 1.035)	.083	0.786 (0.582, 1.062)	.116
**Activity Group**								.033
Inactive (Ref)	–	–	–	–	–	–	–	–
Insufficiently Active	–	–	–	–	–	–	0.998 (0.764, 1.304)	.987
Active	–	–	–	–	–	–	0.759 (0.505, 1.140)	.184
Highly Active	–	–	–	–	–	–	0.453 (0.240, 0.853)	.014

Note: Type III tests were applied to all categorical variables. The corresponding p-values for each categorical variable represent the overall test of significance and are reported in the row of the variable name, not for individual categories.

In Model 2, Sex, Family Income, and Alcohol Consumption were significant.

In Model 3, Sex, Family Income, and Occupation Type were significant.

In Model 4, Sex, Family Income, Occupation Type, and Activity Group were significant.

Table S3. Logistic regression results for Model 4 with MVPA as a continuous variable (n = 1,896).

Variable	Adjusted OR (95% CI)	p-value
PC1	1.050 (0.941, 1.171)	.391
PC2	0.976 (0.877, 1.086)	.651
PC3	1.117 (1.003, 1.244)	.044
PC4	1.008 (0.906, 1.121)	.884
**Continuous Variable**		
Age	1.058 (1.041, 1.076)	<.001
MVPA	0.999 (0.997, 0.999)	.012
**Sex**		<.001
Male (Ref)	–	–
Female	0.403 (0.299, 0.543)	<.001
**Alcohol Consumption**		.075
Non-drinker (Ref)	–	–
≤1 time/week	0.867 (0.635, 1.184)	.368
2–4 times/month	1.087 (0.773, 1.528)	.632
2–3 times/week	1.245 (0.844, 1.836)	.270
≥4 times/week	1.674 (0.994, 2.820)	.053
**Family Income**		.041
1st Quartile (Ref)	–	–
2nd Quartile	0.940 (0.674, 1.311)	.717
3rd Quartile	0.661 (0.465, 0.939)	.021
4th Quartile	0.730 (0.513, 1.038)	.080
**Smoking Status**		.192
Non-smoker (Ref)	–	–
Former Smoker	1.346 (0.968, 1.871)	.077
Current Smoker	1.258 (0.873, 1.814)	.218
**Occupation Type**		.005
Non-manual Worker (Ref)	–	–
Manual Worker	1.117 (0.875, 1.426)	.374
Economically Inactive	1.964 (1.316, 2.931)	<.001
**Work Schedule Type**		.109
Day shift (Ref)	–	–
Evening/Night Shift	0.618 (0.405, 0.944)	.026
Rotating Shift	0.728 (0.392, 1.353)	.316
Other	1.222 (0.285, 5.230)	.787
Not Employed	0.785 (0.581, 1.061)	.115

Note: All variables were mutually adjusted. MVPA was treated as a continuous variable, reflecting total weekly activity time. FPCA scores represent temporal patterns of physical activity. For categorical variables, Type III tests were used to assess overall significance, and the corresponding p-values are reported in the row of each variable name. The results indicated that among the categorical variables, Sex, Family Income, and Occupation Type were statistically significant.
